# Moving Together, Apart: Impacts of Travel Restrictions on a UK–Egypt Collaboration in Health Profession Education

**DOI:** 10.1177/23821205241253668

**Published:** 2024-05-09

**Authors:** Lamis Ragab, Monica Wassim, Zakia Arfeen, Rhiannon Newman, Mohammed Ahmed Rashid

**Affiliations:** 1 School of Medicine, Newgiza University, Cairo, Egypt; 2 Faculty of Medical Sciences, University College London, London, UK

**Keywords:** international, collaboration, pandemic, medical education

## Abstract

**Objective:**

University College London (UCL) and Newgiza University (NGU) have been in an academic collaboration since 2016 to establish undergraduate healthcare programmes in Egypt with an underlying ethos of capacity building and co-development. We explored impacts of pandemic-related travel restrictions on staff across both organisations.

**Methods:**

We conducted 30 semi-structured interviews with academic and professional services staff from UCL and NGU schools of medicine, dentistry, and pharmacy. Data were jointly coded using reflexive thematic analysis and categorised according to the American Council on Education's Comprehensive Internationalisation Framework.

**Results:**

Nine themes were identified, which related to each of the six components of the framework. In addition to mobility, participants’ experiences also spread across the other five components (institutional commitment, leadership, curriculum, faculty support, and partnerships). Successful adaptations were made and staff felt able to ‘keep the show on the road’. However, staff remained keen to keep in-person engagement a priority when possible, especially for quality management site visits.

**Conclusions:**

Travel restrictions can have widespread impacts on all aspects of international collaborations. In this well-established relationship, there was sufficient resilience to withstand these impacts and, many positive unintended consequences emerged. A hybrid engagement model should be prioritised in future partnerships.

## Introduction

Egypt has a long and rich intellectual heritage and over the last century globalisation has influenced the rise and spread of education of varying styles in Egypt. This has partly been, as a result of European professors holding leadership positions at Egyptian universities^1,2^ as well as governmental policies on higher education in Egypt, though these have been mixed in their degrees of success.^3^

The rich historical and political backdrop of higher education within Egypt, provided the background for the conceptualisation of a new university that would ‘challenge the ordinary’. A university that is built on a diverse community of staff and students to spearhead academic and scientific advancement in Egypt and would continue to adapt in future endeavours. Endorsed by Presidential Decree as a non-profit, private university, Newgiza University (NGU) officially opened in 2016 with a foundational focus on healthcare sciences, launching schools in Medicine, Dentistry, and Pharmacy. As part of its mission to create an exciting learning environment that is filled with energy and cultural diversity to redefine the future of Egypt, NGU had a strategic objective to partner with a leading overseas university with a strong track-record in health professions education (HPE) to co-develop these new programmes.

### International partnerships

Although globalisation has been shaping education for many centuries, its impacts on the modern higher education sector have been especially notable in recent decades. In the 1980s, students and faculty members began to travel overseas to seek educational experiences in large numbers, and this was followed in the 1990s by the widespread movement of educational tools and resources in response to the global demand for high-quality education opportunities.^4^

One notable phenomenon has been the delivery of curricula outside of the country in which they were developed. A variety of terms have been used to describe such arrangements, including ‘offshore education’, ‘transnational education’, and ‘crossborder curriculum partnerships’.^5^ The purpose of these partnerships is to provide comparable educational experiences in both institutions and can be attractive for students who are seeking a foreign qualification but for personal or financial reasons do not wish to move overseas.

A literature review on international curriculum partnerships noted that almost all identified articles were about early-stage developments, reflecting that this is a growing and evolving topic.^6^ It found that the most common reason for partnerships to deteriorate is mistrust and ‘disturbed relationships’, going on to conclude that there are no quick fixes to resolve cultural differences and that personal collaborations at all levels should be prioritised. Kosmützky and Putty^7^ note in their systematic review that the academic literature is dominated by single case studies, with little comparative data and crucially, that most of the literature is not from hosting countries. This not only indicates that there are many possible directions for future research but also particularly those that include the views of both parties involved in these partnerships.^7^ Likewise, a systematic review of international branch campuses found ‘a lack of research on a wide range of areas’^8^ highlighting the gap in research here.

Within medical education, Hodges et al^9^ observed the growing trend of international education partnerships within a rapidly evolving globalisation discourse within the field.^9^ There is evidence that there are both financial and academic advantages to both institutions involved in an international education partnerships in medicine,^10^ and another study highlighted that students on three medical school programmes supported by such partnerships were extremely positive about their experiences.^11^

A review article seeking to provide guidance for those establishing international education partnerships, described their tips across four common themes of governance, curriculum, learning environment, and relationship management.^12^ This not only describes the multitude of areas which require careful consideration in an international collaboration, but through relationship management highlight the value of building communities of practice where there is mutual exchange of ideas. A study examining the perspectives of curriculum designers working on international medical programmes, meanwhile, described a graduate profile of a ‘global’ or ‘universal’ physician fit for international practice, which could present tensions with local healthcare contexts and priorities.^13^ There are important cultural differences which need to be considered in international collaborations which may impact curriculum design and delivery such as one study which found opposing viewpoints on the concept of ‘good communication’ between medical students in different continents with Egyptian students having a notable negative perception of ‘western doctors’ emotional aspects of communication.^14^ Although the literature in HPE has been dominated by medicine, there has also been growing interest in international partnerships for other disciplines including pharmacy^15^ and dentistry.^16^

### The University College London–NGU collaboration

From its inception, the stated vision of NGU has been to establish healthcare programmes that inspire and educate a new generation of clinicians by moving away from structures and practices of traditional universities in the region that limit effective learning. The NGU leadership team sought to reduce class sizes, focus on innovation and leadership, and move away from didactic and theoretical teaching, and towards a more patient-centred and practice-focused model of education.

University College London (UCL) is based in the heart of London in the UK and is consistently ranked as one of the world's best universities. Founded in 1826, it was the first university in England to welcome students of any class and religion, and the first to welcome women on equal terms with men. Undergraduate healthcare programmes at UCL are integrated, patient-focused, and aim to equip graduates to thrive as scientifically literate and compassionate clinicians in the constantly changing healthcare landscape. It was these characteristics that led NGU to enter a collaboration with UCL in 2016.

The underlying ethos of this collaboration has been of respectful co-development, with each team mindful of what they bring by way of expertise and experience. Another area of deliberate focus has been on enabling and empowering staff to achieve sufficient mastery and confidence that NGU becomes sustainable with reduced need for support from UCL. The UCL curricula, assessments, and policies have been the basis for the corresponding NGU programmes. Faculty support from UCL with strong collaborative work between project teams alongside regular steering group meetings have allowed for the adaptation and contextualisation of these to fit with the vision and operating environment of NGU.^17^

Although geopolitical unrest such as social and political tensions and military conflict which had occurred in Egypt in the past was identified as a risk by both parties at the outset, this had not affected the project thus far. Instead, it has been a communicable disease outbreak, COVID-19, that has proved to be the greatest disruption. The widespread impacts of COVID-19 on universities and specifically HPE have been well rehearsed.^18,19^ Restrictions to air travel and international movement meant that for almost two years, the UCL-NGU collaboration shifted to an entirely virtual format. Prior to this, regular annual visits of each team to the partnering institution would occur though events and workshops, building on face-to-face networking, collaborative working and direct faculty development. This period of physical distance between the respective teams dramatically and unexpectedly changed the course of the collaboration where all educational aspects of the partnership that had been established continued and where actively maintained. This meant that while in-person visits had to stop, there was an increase in the use of Virtual Meeting Platforms such as MS Teams and email communication alongside regular steering group meetings with adjusted and flexible agendas. Given the established importance of relational factors in international partnerships,^20,21^ we sought to use the natural experiment offered by these travel restrictions to explore their impact on the UCL-NGU collaboration. Our research question was therefore ‘What are the experiences and reflections of medical educators on the impact of travel restrictions on a UK-Egypt collaboration in health profession education’.

## Methods

We undertook this research between March and August 2022 when travel restrictions were beginning to be lifted globally. At this stage, no UCL or NGU staff had been able to meet in person for over two years since early 2020. Given the focus on capacity building, the collaboration was primarily designed to support the NGU faculty to teach NGU students directly. The UCL team has therefore primarily played a ‘back room’ role in helping to support NGU staff in all aspects of their planning and delivery, and to co-develop NGU curricula and assessments using UCL materials as a basis.

In this qualitative study, we took an interpretive methodological perspective which acknowledges the subjective and co-constructed nature of knowledge to develop rich insights based on our research question.^22^ Given the importance of personal and relational elements,^23^ in-depth interviews were used to explore individual experiences and reflections. Participants were recruited by direct email correspondence in a purposive manner to ensure coverage across the UCL and NGU schools from each of the three schools (medicine, dentistry, pharmacy), as well as coverage across different levels of seniority, duration of engagement with the collaboration project, and across the academic and professional services spectrum. The inclusion criteria used for this study is shown in Table 1.

**Table 1.** Inclusion criteria for participants in the study.

1. All participants that were current members of faculty either at University College London or NewGiza University were eligible to participate
2. All participants belonged to either the school of Medicine, Dentistry or Pharmacy
3. All participants had been actively involved in the collaboration between UCL and NGU
4. All participants had been involved in the collaboration both prior and during the pandemic.

All participants that were invited were involved and written informed consent was obtained using forms and verbal consent was reaffirmed prior to starting. Semi-structured interviews were used (see appendix) in this study which allowed for both open-ended and focussed questioning. This was piloted with two participants from each institution (13% of all interviews) who provided feedback and no changes were made to the questions. The questions covered the participants’ experiences and thoughts on what the partnership programme was like pre-COVID 19, how the partnership programme responded to COVID-19 and what implications resulted from changes that were implemented in response to COVID-19. All interviews were conducted via Microsoft Teams videoconferences between the participant and interviewers only and transcribed using detailed field notes. No repeat interviews were required and participants were not involved in transcription review or feedback on findings. The field notes formed the basis of the data which were subsequently coded iteratively and interrogated jointly by the cross-institutional research team using thematic analysis and data saturation was reached. The study was approved the University College London Research Ethics Committee (15443/006) and the Newgiza University Research Ethics Committee (N-12-2021).

The team approached the data with a reflexive thematic analysis lens,^24^ where existing knowledge about collaboration responses could be acknowledged and considered in the interpretation. Field notes were read extensively and repeatedly to become familiar with the content and then coded individually by researchers from the respective countries. The team then reviewed these initial themes and concluded that they related to one of the six pillars of the American Council on Education (ACE) Comprehensive Internationalisation Framework (Leadership, Curriculum, Faculty support, Mobility, Partnership, Institutional commitment).^25–27^ A thematic framework was devised using these six pillars. Two authors (MAR and MW) independently reviewed the codes again to ensure the framework captured the breadth of topics covered in transcripts. Final coding was agreed by all authors and consensus meetings were used to resolve areas of disagreement.

The author team was deliberately mindful of how their own experiences and positions influenced the research. The entire research team had been involved in the UCL–NGU collaboration and was all staff at one of the two universities. However, interviews were conducted by two female researchers (MW and RN) who had played a peripheral role in the collaboration and were not involved in its design, leadership, or governance and had previous experience in qualitative research. The author team varied in seniority from teaching assistant (MW) to clinical lecturer (ZA and RN) and professor (LR and MAR) and had a variety of previous experiences in HPE and international partnerships.

## Results

A total of 30 interviews were conducted, comprised of 10 with UCL staff and 20 with NGU staff. Participants were relatively evenly split across the 3 healthcare courses (pharmacy = 8, dentistry = 11, medicine = 11). Twenty-one participants were academic staff which included academic subject leads and clinical lecturers and 9 were professional services staff, which were predominantly project managers involved in administration of the collaboration alongside project administrators and business managers. The interviews were conducted between March and August 2022 and lasted between 45 and 60 minutes in duration.

Nine themes were identified in our analysis, which are listed in Table A1, categorised against the six pillars of the ACE Comprehensive Internationalisation Framework, with illustrative quotes for each theme. These are described in turn below.

### Leadership

The importance of engagement from senior leadership teams was highlighted by participants from both universities. From the outset of the collaboration, each of the three schools established regular calls that acted as a ‘touchpoint’ as quoted by participants, for teams on either side to connect and provide updates. Importantly, since the outset of the collaboration, the deans of the three NGU schools and their counterpart directors of the three UCL departments took part in these team meetings. In two schools, the directors eventually passed on operational leadership to senior academic colleagues but remained intimately involved in the project. This high level engagement was deemed significant by participants as it expressed the importance given to the collaboration. This dialogue between senior decision makers on each side about how to respond and adjust to restrictions played an additional crucial role when the pandemic hit. Participants noted that in the fast-changing and rocky environment that they faced, having senior leaders remain connected with institutional, and even national, policy-making, despite not being able to travel was extremely beneficial to help keep the collaboration on track.

### Curriculum

It was clear to all participants that the pandemic represented a once-in-generation event that posed serious threats to healthcare education. In this uncertain and stressful climate, participants felt reassured by sharing experiences with their collaborators, giving a sense of the shared challenge. Participants found themselves comparing and contrasting how their own countries and societies, as well as their own universities and students, were responding to the various restrictions placed on them, especially in fields that rely heavily on in-person learning through placements and clinical settings. Although major curricula shifts were determined by national policy, participants nonetheless valued regular online discussions about how these adaptations could best be operationalised. A further theme linked to curriculum was the advent of remote teaching of clinical topics, which had not previously been part of the collaboration. As faculty members became comfortable with online teaching, the step to teaching across institutions felt more manageable and was taken up by all three schools. There was broad consensus by participants that this was a positive unintended consequence of being forced apart by travel restrictions and one they were keen to maintain.

### Faculty support

Prior to travel restrictions commencing, in Spring 2020, a UCL team were due to visit NGU to conduct staff training. The plans changed at a very late stage and there was disappointment from both teams that this could not go ahead. However, as it became clear that the restrictions would go on for months and years rather than days and weeks, each of the three schools designed online training programmes. These typically mimicked timetables from the previous in-person training events, including tasks to be completed in between sessions and days of the programme. These events were welcomed, and participants noted they could form the basis for a more flexible training approach and as stated by one participant ‘opens our minds about what is possible’ to explore new and novel approaches in an already established partnership. As well as group training events, professional services staff from both sides acknowledged the additional individualised training support that became possible through platforms such as Microsoft Teams. In particular, the ability to share screens and work collaboratively on education planning documents was valued.

### Mobility

Adapted communication channels such as videoconferencing platforms (Zoom and Microsoft Teams) that were adopted to mitigate travel restrictions were largely met positively. Despite this, though, on reflection of the nature of the collaboration during the pandemic, participants recognised there were unique benefits of in-person engagement. It was considered, therefore, that travel should be deemed ‘postponed’ rather than cancelled altogether, and there was enthusiasm for reinstating travel when possible. Quality assurance visits were consistently highlighted as an area where in-person travel would be beneficial to allow detailed evaluation of the evolving NGU programme. It was also hoped that some in-person faculty development could also return, especially in practical and clinical education areas.

### Partnerships

An important transformation that occurred in the early phase of pandemic-related disruption was for the collaboration to switch from teleconferencing to videoconferencing as the primary communication channel. This was unanimously popular amongst participants. One unexpected benefit identified was that it allowed a more human connection to be established as participants felt interactions were enriched, not only by seeing each other visually, but also by seeing people's background locations and settings, especially when working from home. There was also a recognition that travel restrictions and the broader pandemic upheaval provided an opportunity to critically reflect on the nature of the collaboration. Some participants noted that there was a tendency to assume that the UCL approach was superior or correct because of its geopolitical position, history, and reputation. One participant states ‘We need to reflect on what is western and what is best*’* which indicates a challenging of presumed hierarchies of power and status. This is particularly interesting in terms of recognising power imbalances and decision making between the institutions which although not specifically explored, does indicate that participants felt that there was a perceived balancing in these differentials compared to previously due to the imposed restrictions.

### Institutional commitment

Although travel restrictions caused a complete stop on any in-person movement in either direction, the collaboration remained important to both partners and continued uninterrupted despite the often chaotic backdrop. It was noted that despite busy schedules, including of clinical staff who had professional commitments in the healthcare service, there was no pause or slowdown of the collaboration. Participants from both institutions reflected on the powerful message this sent. In fact, the need to realign and recalibrate approaches through this uncertain period allowed more opportunities for cross-departmental meetings in a way that may have been more logistically difficult previously and did not happen before. Participants noted that they were able to ‘work flexibly and remotely’ and in remote situations ‘where we always thought we couldn’t’ highlighting the concept that both institutions continued and can continue to work together, despite being apart.

## Discussion

This study examined impacts of travel restrictions that caused significant disruption four years into an international education collaboration between healthcare faculties in an established UK university and a brand new one in Egypt. On the inception of this collaboration, travel of key members of faculty was seen as a central aspect to the development and operationalisation of a successful partnership between two distinct institutions in far-reaching countries. Initially, the influence of these restrictions seemingly affected mobility, which is just one component of the ACE comprehensive internationalisation framework, however we found that those involved in the collaboration described widespread impacts across all six areas of the framework. This reaffirms the interconnected nature of the framework and underlines the key importance of travel and in-person engagement as enabler of effective international education partnerships.

The existing literature on adaptations made through pandemic restrictions has emphasised the role of technology in enabling solutions to overcome challenges posed by non-pharmacological interventions such as social distancing.^28–30^ We found that videoconferencing platforms enabled both closer engagement between staff and the ability to conduct remote teaching across institutions. Importantly, though, participants in the UCL–NGU collaboration emphasised the relational and socioemotional implications of these technologies as key to their experiences as opposed to technical factors. For example, opportunities to connect on a more human level and consider power dynamics. This aligns with a growing literature that has recognised that interpersonal factors are the most important when collaborating in international medical education.^31,32^

A noteworthy finding in our study is that despite the success of various remote and virtual engagements, there remained a keenness to keep opportunities for in-person engagement in the future of the collaboration. Quality assurance visits were particularly highlighted as an area that would benefit from in-person site visits. This fits with the recent higher education literature, where caution has been urged when it comes to digitisation of quality assurance activities to ensure sufficient priority is given to maintaining integrity.^33,34^

The strengths of this work include the reflexive thematic analysis by a team of cross-institutional researchers with a deep understanding of the context of each university, as well as the application of the ACE comprehensive internationalisation framework to understand these findings in the broader higher education landscape. Although in-depth interviews allowed for nuanced and detailed explorations, the study was limited by this being the sole method of data collection. A further limitation concerns the participants being limited to core collaboration teams at each university, which may not have captured the views of those involved more peripherally, or those only indirectly involved in, or impacted by, the collaboration.

This study has a number of implications for those working in international medical education partnerships. Firstly, it highlights the complex operating environments that they represent, whereby an apparently single issue of reduced mobility and travel restrictions has profound impacts on the entire enterprise. Secondly, it reiterates the existing literature on relational factors and communication being a core element of international education partnerships. Thirdly, it suggests that despite some elements of collaboration activities working well in a virtual format, there are aspects which collaborating staff clearly feel should continue with in-person engagement when safe and appropriate to do so. In sum, these findings suggest that senior leaders responsible for setting policies around international education partnerships should prioritise a hybridised model that incorporates a mixed mode of engagement.

Further research in this area could focus on how findings linked specifically to international collaborations intersect with the emerging literature about the impact of travel restrictions to staff mental illness and wellbeing.^35^ Additionally, our finding that quality assurance visits benefit from in-person visits is worthy of further exploration to understand the socio-spatial relations of making quality judgments in HPE. Finally, the recognition in this study of Western practices being considered superior aligns with a growing interest in applying postcolonial theory to global medical education, especially during crises and although it was not central to our own findings it should be researched further.^36–38^

## Conclusion

International education partnerships in healthcare are an area of growing popularity and scholarly interest. The collaboration between UCL and NGU was in a stage of reaching stability and maturity when it faced an existential threat posed by pandemic-related restrictions to international travel. This study shows that staff involved in the collaboration adapted and ultimately, the work persisted effectively with many innovations and unintended positive consequences emerging. The academic collaboration between institutions has been of mutual benefit throughout its course and this study has highlighted some important considerations for those considering starting a new partnership, where the aim is to develop, address challenges and move together, despite being physically apart. Importantly, the experiences of staff involved in this collaboration highlight that the impacts of reduced mobility was extensive, as demonstrated by the spread of data themes across the components of the ACE Comprehensive Internationalisation Framework. Despite the many successes of this period, the keenness of participants to resume in-person engagements suggests that the future of international education partnerships is likely to need a flexible and hybridised approach.

## Appendix

Table A1 Themes with illustrative quotes

**Table:**

ACE comprehensive internationalisation framework component	Theme	Illustrative quote
Leadership	Senior engagement	We were lucky that the relationship was already established between our respective senior leadership teams
Curriculum	Remote teaching	UCL staff delivered a series of online live tutorials to NGU students directly… we can now support NGU in a more cost and time effective manner without travelling
	Shared experiences	Both institutions were firefighting in their own backyards. It was a shared, global experience
Faculty support	Online training	The online staff training went well, and it was helpful to cover the examinations
Mobility	Postponement	If the pandemic is more short term then the relationship is not endangered… Some elements work better on site
Partnership	Humanisation	We became more familiar with each after having online calls… we could also have some small talk, such as talking about colleagues’ cats!
	West isn’t best	We need to reflect on what is western and what is best. We thought these topics are a poverty issue not a global issue, how wrong were we?!
Institutional Commitment	Business as usual	The core focus of work was providing input in teaching material, assessments and we continued very much in the format as before
	Internal reconnections	As well as our relationships with our partners, our own relationships with colleagues in the other healthcare schools was strengthened through this collaboration

### Semi-structured interview guide – version date June 14, 2021

Examining impacts of COVID-19 on established international capacity building relationships in medical education.

### Introduction

Thank you for agreeing to participate in today's interview. The purpose of our discussion today is to better understand how the COVID-19 pandemic has influenced your partnership over the past 18 months. Specifically, we are hoping to identify how your program has responded to COVID-19, the modifications that you’ve had to make within your program, and the implications of these modifications for you, the faculty within the program, and the students enrolled in the program.

### Questions and Consent

Before we start the interview, I’m wondering if you have any questions about this research?

[Interviewer to clarify any questions and ensure that consent has been obtained].

Section 1: What was the partnership program like pre-COVID-19?

1. What was the structure of your partnership program like before 2020?

o How many courses, visits took place?

o What was the format of the courses/modules?

o How many faculty taught within the program and how often?

2. What was your role within your partnership program?

o How often did you teach in the program?

o How were you involved in making decisions about course content, format, structure?

o How did your role differ from the roles of others within your partnership program?

3. What underlying principles and values directed your partnership program?

o Who determined the goals and objectives of the partnership program?

o How did individuals communicate and collaborate in identifying goals and objectives?

o How were tensions related to conflicting principles, values and goals resolved?

Section 2: How did the partnership program respond to COVID-19?

4. What were the primary changes that were made to your partnership program in response to COVID-19?

o Were any changes made to the structure of the program?

o Were any changes made to the format of the program?

o Were any changes made to the content of the program?

5. How were decisions made about how to best respond to COVID-19?

o Who was responsible for making these decisions?

o What kinds of considerations were accounted for in making changes?

o What were the most essential aspects of the program that you strived to maintain?

Section 3: What implications resulted from changes that were implemented in response to COVID-19?

6. What outcomes resulted from the changes that were implemented within your partnership program?

7. Were there any unintended consequences from the changes that were implemented within your partnership program?

8. Do you have any concerns about the long-term consequences of these changes?

9. Have any of the changes that were implemented in response to COVID-19 led to concerns about issues of equity and power dynamics within your partnership program?

10. Upon reflection, are there other ways that you think your partnership program could have responded to COVID-19?

11. Moving forward, how do you envision your partnership program functioning as COVID-19 continues and as we reach a state of resolution?
